# Use of rhodamine B to mark the body and seminal fluid of male *Aedes aegypti* for mark-release-recapture experiments and estimating efficacy of sterile male releases

**DOI:** 10.1371/journal.pntd.0005902

**Published:** 2017-09-28

**Authors:** Brian J. Johnson, Sara N. Mitchell, Christopher J. Paton, Jessica Stevenson, Kyran M. Staunton, Nigel Snoad, Nigel Beebe, Bradley J. White, Scott A. Ritchie

**Affiliations:** 1 College of Public Health, Medical and Veterinary Sciences, James Cook University, Cairns, Australia; 2 Australian Institute of Tropical Health and Medicine, James Cook University, Cairns, Australia; 3 Verily Life Sciences, South San Francisco, CA. United States of America; 4 School of Biological Sciences, University of Queensland, St Lucia, Brisbane, Queensland, Australia; 5 Commonwealth Scientific and Industrial Research Organisation, Dutton Park, Queensland, Australia; The Pennsylvania State University, UNITED STATES

## Abstract

**Background:**

Recent interest in male-based sterile insect technique (SIT) and incompatible insect technique (IIT) to control *Aedes aegypti* and *Aedes albopictus* populations has revealed the need for an economical, rapid diagnostic tool for determining dispersion and mating success of sterilized males in the wild. Previous reports from other insects indicated rhodamine B, a thiol-reactive fluorescent dye, administered via sugar-feeding can be used to stain the body tissue and seminal fluid of insects. Here, we report on the adaptation of this technique for male *Ae*. *aegypti* to allow for rapid assessment of competitiveness (mating success) during field releases.

**Methodology/Principle findings:**

Marking was achieved by feeding males on 0.1, 0.2, 0.4 or 0.8% rhodamine B (w/v) in 50% honey solutions during free flight. All concentrations produced >95% transfer to females and successful body marking after 4 days of feeding, with 0.4 and 0.8% solutions producing the longest-lasting body marking. Importantly, rhodamine B marking had no effect on male mating competitiveness and proof-of-principle field releases demonstrated successful transfer of marked seminal fluid to females under field conditions and recapture of marked males.

**Conclusions/Significance:**

These results reveal rhodamine B to be a potentially useful evaluation method for male-based SIT/IIT control strategies as well as a viable body marking technique for male-based mark-release-recapture experiments without the negative side-effects of traditional marking methods. As a standalone method for use in mating competitiveness assays, rhodamine B marking is less expensive than PCR (e.g. paternity analysis) and stable isotope semen labelling methods and less time-consuming than female fertility assays used to assess competitiveness of sterilised males.

## Author summary

The recent resurgence in male-based “rear and release” strategies (release of radiation sterilised males or those harbouring *Wolbachia*) to control diseases transmitted by urban *Aedes* mosquitoes, including dengue, chikungunya, and Zika, has revealed the need for an economical and rapid diagnostic tool to assess the mating competitiveness of released males against wild counterparts for receptive females. Our report details the optimisation of rhodamine B labelling of male seminal fluid and visible body staining via sugar-feeding with no significant impact on survival or mating competitiveness, as well as proof-of-principle field releases. These results reveal rhodamine B labelling to be an economical and rapid evaluation method for male-based control strategies as well as a viable body marking technique for male focused mark-release-recapture experiments without the negative side-effects of traditional marking methods. The use of this method has the potential to enable operators to determine the efficacy of their control method quickly and easily, allowing them to optimise releases to achieve optimal population suppression.

## Introduction

Knowledge of key entomological parameters that contribute to the transmission of mosquito-borne diseases such as dispersal range, blood-feeding rates, population size, survival, and length of gonotrophic cycles is invaluable for understanding disease transmission dynamics and for determining the extent of control measures necessary to interrupt transmission [1–5]. Traditionally, much of this information has been obtained by conducting mark-release-recapture (MRR) field experiments, during which the target insect is collected either from laboratory colonies or from the field, marked, released into the field, and recaptured at given time and distance intervals after their release [6, 7]. The recaptured insects are then checked for the presence of the marker to distinguish them from unmarked individuals. Although a variety of methods have been used to mark mosquitoes, including dyes, paints, trace elements, and radioactive isotopes, the current “gold-standard” marking technique for mosquito vectors is the use of fluorescent dusts (also commonly referred to as powders) to externally mark the body of released individuals (for review, see [6–8]). These dusts are available in a wide range of colours and are easily applied and detected with or without inexpensive UV lights. This method in one form or another has been used to mark insects for > 70 years [9], including mosquitoes belonging to the genera *Aedes*, *Anopheles*, *Culex*, as well as many others [7]. Despite its simplicity, the use of these dusts can increase mortality, decrease mobility and affect the sensory organs, in addition to poor persistence and potential transference to unmarked individuals, giving biased results in MRR studies [8, 10]. The use of dyes and paints can have similar impacts [11–13], whereas the use of protein and isotope labelling can be time consuming, expensive and require advanced analysis [14–16]. Thus, the development of alternative methods that are cheap, easy to detect, and have minimal fitness costs is needed.

Although the usefulness of MRR experiments is well known, for mosquitoes they have overwhelmingly focused on assessing female dispersal and survival due to their importance in disease transmission. A recent review of MRR experiments involving *Aedes*, *Anopheles*, and *Culex* mosquitoes revealed 774 out of 800 reports focused on adult females [7]. Consequently, much less is known of male survival, dispersal, and population dynamics in general. However, renewed interest in male-based sterile insect technique (SIT) control programs, including those reliant on radiation [17], genetic modification [18, 19], and the use of the *Wolbachia* bacteria to induce male sterility (incompatible insect technique (IIT) [20])), has revived interest in understanding male ecology. This is knowledge best attained through MRR experiments, and because the goal of SIT/IIT programs is to reduce mosquito populations and disease transmission, they would benefit from a marking technique that allows them to quickly and easily assess the efficacy of their method. Specifically, SIT/IIT programs would benefit from a marking technique that allows for marking of the male body and, more importantly, one that marks the seminal fluid or sperm to enable researchers to assess the mating success of their released males with wild females quickly and affordably. This would allow operators to obtain better estimates of intervention efficacy and enable them to alter operations accordingly in real-time. Such a technique may be achieved by simply feeding males on sugar solutions containing a fluorescent dye, rhodamine B, to stain the seminal fluid and body tissue. Rhodamine B is a thiol-reactive fluorescent dye prized for its photostability and solubility that forms covalent bonds to proteins. It produces a red-violet staining of tissue and emits a distinct bright red colour when fluoresced (excitation maximum 540 nm, emission maximum 625 nm). It has proven to be particularly useful for marking insects, for example, when administered via sugar-feeding it has been used to assess mating events in tobacco budworm moths [21], fireflies [22, 23], and *Drosophila* [24], as well as the successful staining of the body of female *Culex* mosquitoes [25]. Although rhodamine B marking appears to have no effect on mating behaviour, a shortened lifespan of *Photinus* fireflies after feeding has been reported [22]; however, the same was not observed for *Culex* mosquitoes [25] or moths [21]. These observations, combined with a minimal chance of damaging specimens or of cross-contamination by body contact, suggest rhodamine B labelling may be advantageous to fluorescent powders and an extremely useful marking technique to assess male mating success.

Thus, the main objective of this study was to assess the potential of rhodamine B as a dual marking technique for male *Ae*. *aegypti*, including a *Wolbachia*-infected (*w*Mel) strain due to recent releases of *Wolbachia-*infected *Ae*. *aegypti* and *Ae*. *albopictus*, for either its virus-blocking capabilities [26], sterilization by cytoplasmic incompatibility [20], or both [27]. Specifically, we report on a series of experiments with the goals of determining optimal feeding regimes for ideal body and seminal fluid marking, as well as any potential impacts of rhodamine B feeding on male survival and male mating competitiveness. We conclude by presenting results from proof-of-principle field releases.

## Materials and methods

### Mosquitoes

All mosquito colonies were maintained using standard laboratory rearing protocols [28, 29]. Wild type *Ae*. *aegypti* were sourced from routine ovitrap collections from multiple locations in Innisfail, QLD, Australia in 2016. Field collected ovistrips were hatched, larvae sorted to species, and reared to adults. Adult females were then blood-fed and allowed to oviposit, after which the eggs (F1 generation) were stored at 28°C and 70% RH until needed. A colony of *w*Mel (*Wolbachia*) infected *Ae*. *aegypti* was established from ovistrip collections from multiple locations in Cairns, QLD in 2016. This colony was continually supplemented from additional ovitrap collections leading up to the experiments, but has been maintained at a moderate population size of a couple hundred individuals since establishment. Laboratory and semi-field cage experiments were conducted at the James Cook University Mosquito Research Facility in Cairns, QLD. Laboratory experiments were conducted at 28° C and 70% RH, whereas temperatures in the semi-field cage ranged from ∼25° C in the colder months (May-September) to ∼30 C during the hotter months (October-February). Field studies were conducted at a single Queenslander style residence located in the Edge Hill suburb of Cairns, QLD from August-September 2016.

### Rhodamine B feeding

Wild type and *w*Mel *Ae*. *aegypti* males were fed on 0.1, 0.2, 0.4, or 0.8% (w/v) rhodamine B (Sigma Aldrich, 95% dye content, HPLC) dissolved in a 50% honey solution (diluted in distilled water). Males had free access to the solutions via a sponge (3 x 3 x 0.5 cm) holding ~5 ml of solution placed on top of a 70 ml collection jar lid that was placed inside a small cage (30 x 30 x 30 cm; BugDorm, MegaView Science Co., Ltd.) and rewetted every two days. Mosquitoes were held at 28° C, 70% RH, and on a 12/12 h L: D cycle during feeding. To ensure virginity prior to being placed into the cages, males and females were separated during the pupal stage individually according to size and kept in cups (720 ml) containing 10–20 pupae until emergence without sugar. The cups were then inspected to ensure that no females were present before males were released into the cage. All males were 12–24 h old at the start of each experiment.

### Experiment 1: Effect of concentration and feeding time on rhodamine B body and seminal fluid marking

**Fig 1:**
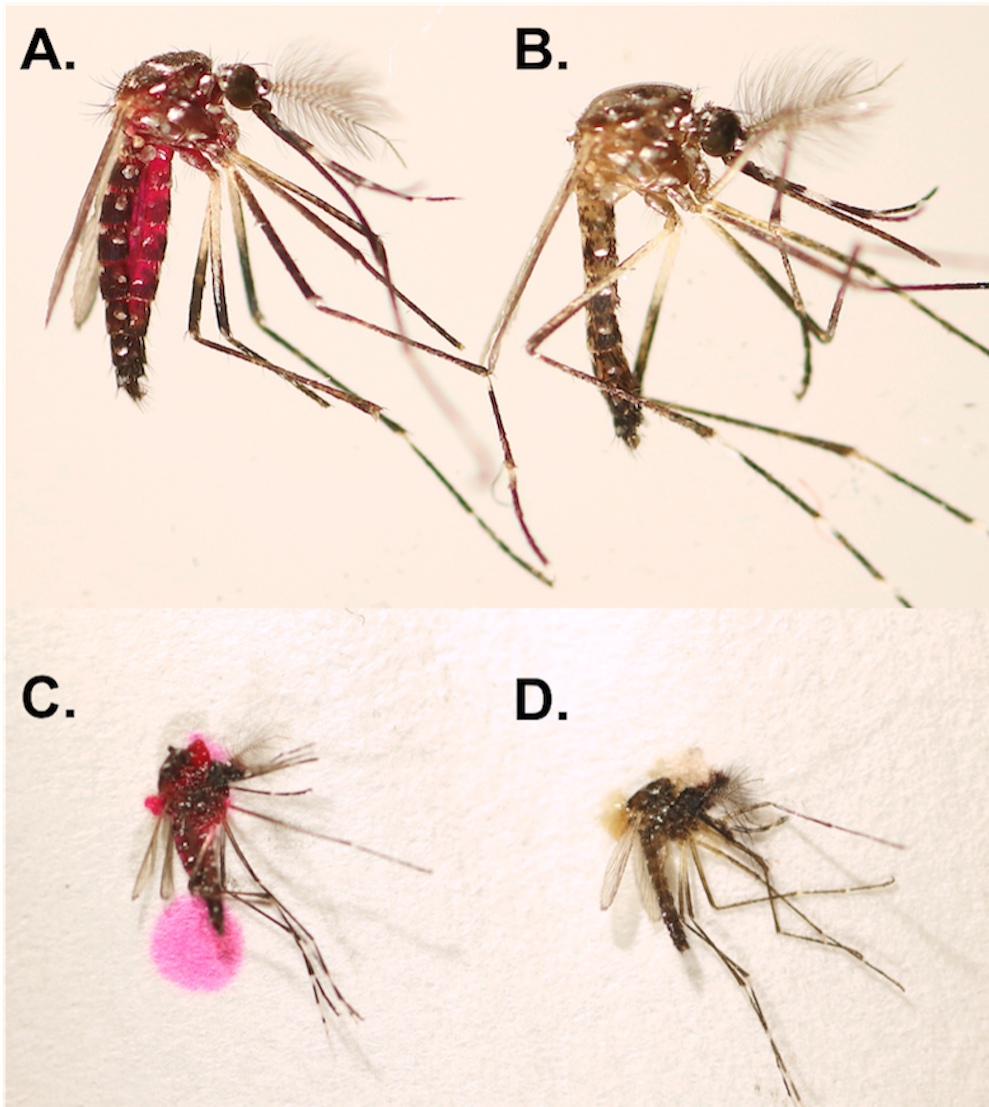
Rhodamine B male body marking. (**A**) Example of rhodamine B body marking of male *Ae*. *aegypti* compared to an (**B**) unmarked male. (**C**) Detection of rhodamine B in a marked male by crushing on filter paper compared to a (**D**) crushed unmarked male. Rhodamine B marked males were fed on a 0.4% (w/v) rhodamine B honey (50%) solution for 4 d.

**Fig 2:**
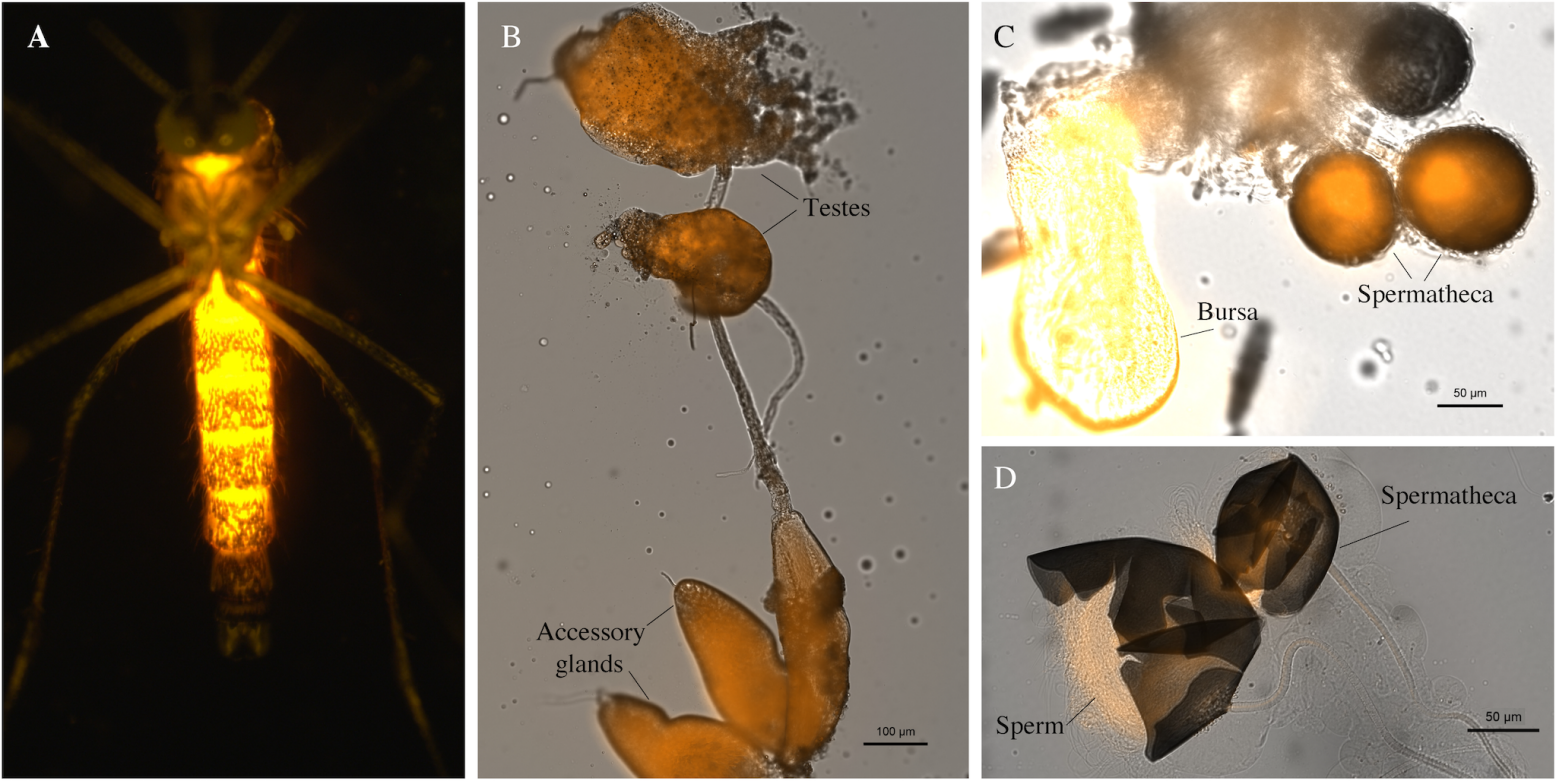
Rhodamine B marking of male seminal fluid. (**A**) Example of rhodamine B fluorescence through cuticle of the abdomen of a male *Ae*. *aegypti*. (**B**) Rhodamine B labelling of the testes and accessory glands of male *Ae*. *aegypti*. (**C**) Rhodamine B labelled seminal fluid in the spermathecae and bursa of an *Ae*. *aegypti* female transferred during mating with a marked male. (**D**) Enlarged view of rhodamine B labelled seminal fluid in the spermathecae of an *Ae*. *aegypti* that have been opened to reveal the presence of sperm. Males in images A and B were labelled by rhodamine B feeding on a 0.2% w/v solution for 4 days and in image C and D were labelled by rhodamine feeding on a 0.4% w/v solution for 4 days.

To obtain information on the minimum number of days necessary to adequately mark the body (conspicuous visual staining) and seminal fluid of males (successful transfer to females) via rhodamine B feeding, the following experiment was conducted. Twenty wild type males were placed in a cage containing either a 0.1, 0.2, 0.4 or 0.8% rhodamine B solution for a period of 1, 2, 3, or 4 days. Male body staining was determined by the visual presence of a dark to bright red colour in the body, primarily in the abdomen and thorax (Fig 1). Staining of seminal fluid was determined by successful transfer to females during mating as determined by the presence of rhodamine B in the bursa, spermathecae or both under fluorescence (Fig 2). To do so, at the end of each time point, twenty 3–5 d old virgin females were introduced into each cage and the rhodamine solution removed and no additional sugar solution added. The mosquitoes were left to copulate for 24 h, after which the mosquitoes were frozen at -20° C for 1 h, then dissected to remove the bursa and spermathecae. The bursa and spermathecae were dissected into a 1% PBS solution and then placed on a microscope slide containing a drop of fluorescent preservative containing DAPI (Fluoroshield with DAPI, Sigma Aldrich) to preserve the fluorescence of rhodamine B and help visualise the presence of sperm via DAPI staining. Once placed on the slide, the sample was gently crushed with a cover slip. The presence of rhodamine B was examined using a fluorescent microscope (Nikon Eclipse C*i*) provisioned with an illuminator (Nikon Intensilight C-HGFI) and a G-2A long pass filter (absorption 510 to 600 nm/emission 575 nm). This filter optimised visual differentiation of rhodamine-treated compared to untreated testes/MAGs (Fig 2). Images were electronically captured using a Nikon DigiRetina 16 camera and related software. A Nikon DAPI filter cube (excitation 340 nm/emission 488 nm) was used to help determine the presence/absence of sperm. Three replicates were performed for each treatment.

### Experiment 2: Effect of rhodamine B feeding on male survival

To obtain information on the effect of rhodamine B feeding on male survival, the following experiment was conducted. Twenty males of either wild type or *w*Mel *Ae*. *aegypti* were placed in a cage containing either a 0.1, 0.2, 0.4 or 0.8% rhodamine B solution or a 50% honey control for a period 4 days, the period which produced the greatest body and seminal fluid marking. At the end of day 4, the rhodamine and honey solutions were removed and no additional sugar solution was added. The number of dead mosquitoes was recorded daily after removal of the sugar solution until all males had died. Two replicates were performed for each treatment.

### Experiment 3: Persistence of rhodamine B labelling of male body tissue and seminal fluid

To obtain information on the longevity of rhodamine B marking of the body and seminal fluid, the following parallel experiments were conducted. In the first experiment, twenty males of either wild type or *w*Mel were placed in a cage containing either a 0.1, 0.2, 0.4 or 0.8% rhodamine B solution for a period 4 d, the period which produced the greatest body and seminal fluid marking. After 4 days of labelling, the rhodamine solutions were removed and no additional sugar solution was added. Virgin females (3–5 d old) were then introduced into subsets of cages 24, 48, and 72 h after removal of the rhodamine solutions and allowed to copulate with males from each labelling regime for a period of 24 h, after which transfer of marked seminal fluid to females was scored as described above. The number of virgin females introduced into each cage was dependent on the number of surviving males maintaining a 1:1 female/male ratio at each introduction due to the high mortality of males beyond 48 and 72 h. The second experiment investigated the longevity of male body marking and consisted of the same experimental setup with the exception that no females were introduced. At the end of each time point (i.e. 24, 48, and 72 h after removal of rhodamine solutions) males were knocked down by freezing at -20° C for 30 min, then visually inspected for the presence of rhodamine B. Males were scored visually for the presence of a dark red to bright red colour in the body and by dissecting out the digestive tract in a 1% PBS solution and determining the presence/absence of rhodamine under fluorescence. Three replicates were performed for each treatment during both experiments.

### Experiment 4: Effect of additional sugar-feeding on male body and seminal fluid marking

To understand the effect of additional sugar-feeding rhodamine B marking of the body and seminal fluid, the following parallel trials were conducted. For trial one, 12 replicates of 15 wild type males were placed in plastic cylindrical containers (11 cm wide x 10 cm deep) with 1 cm of water in the bottom and exposed to 0.4% rhodamine B solution for 4 days. After this period, the solution was removed and the males were starved for 48 hours. Following this period, six randomly selected replicates were exposed to additional sugar-feeding (50% honey solution) for 12 hours and then 12 hours later all males were knocked down, by freezing at -20° C for 30 min, and visually inspected for the presence of rhodamine B. Males were scored visually for the presence of a dark red to bright red colour in the body and by dissecting out the digestive tract in a 1% PBS solution and determining the presence/absence of rhodamine under fluorescence.

The second trial involved 130 males individually placed into identical plastic containers as trial one and exposed to 0.4% rhodamine solution for four days. Like trial one these males were also subsequently starved for the following 48 hours, then half the group (65 randomly selected males) was allowed to feed on 50% honey solution for 12 hours to simulate a nectar feeding in the wild. After an additional 12 hours, a virgin female (of identical age to the males and only fed on 50% honey solution) was introduced into each of the male’s containers for the next 24 hours to facilitate mating. After these events (96 h after removal of the rhodamine B solution), mosquitoes were knocked down and males inspected for the presence of rhodamine B as described in trial one. The females were inspected for rhodamine B in the bursa and spermathecae, as well as the presence of sperm as described above.

### Experiment 5: Mating competitiveness of rhodamine B marked males under semi-field conditions

We performed a series (n = 6) of free-flight mating competitiveness assays within large semi-field cages (17.5 m x 8.7 m x 4.1 m and featuring a simulated house and yard [30]) between 0.4% rhodamine B marked (fed 4 d) and unmarked males to assess the effect of rhodamine B feeding on male mating competitiveness. To ensure that males cannot transfer rhodamine B to previously mated females, rhodamine B marked males (fed 0.4% solution for 4 d; n = 40) were introduced to females (n = 40) exposed to unmarked males (n = 40) for 72 h. After 48 h in the presence of rhodamine B marked males, no transfer of rhodamine was observed and all females were positive for sperm, revealing no transference of rhodamine to previously mated females. For each competitiveness assay replication, we placed 80 virgin females (3–5 d old), 40 virgin marked males, and 40 virgin unmarked males (4–5 d old) in a semi-field cage. 72 h later, females were collected using a Prokopack aspirator [31] and dissected to determine the presence/absence of rhodamine B as described above. These assays were performed for both wild type and *w*Mel *Ae*. *aegypti*. A female was considered mated by a rhodamine B marked male by the presence of rhodamine B in the bursa, spermathecae, or both, as well as the presence of sperm. A female was considered mated by an unmarked male by the presence of sperm without rhodamine in the bursa and spermathecae.

### Experiment 6: Rhodamine B transfer to females during a field release

A series (n = 3) of proof-of-principle field releases were conducted to determine if rhodamine B transfer from marked males to receptive females occurs under field conditions. For each experimental replicate, 150 marked *w*Mel *Ae*. *aegypti* males (0.4% rhodamine B, fed 4 d) were released at 08:00 in the front of a single residence located in the Edge Hill suburb of Cairns, QLD where *w*Mel *Ae*. *aegypti* have been established since 2012 [32]. Because the releases occurred late in the dry season, a period during low *Ae*. *aegypti* abundance [33], we released a total of 30 virgin *w*Mel *Ae*. *aegypti* females (3–5 d old) inside the house 24 h prior to the release of males. Indoor releases were conducted to determine if marked males released outside would enter houses, the preferred resting site of female *Ae*. *aegypti* [34]. Mosquitoes were sampled indoors by 10 min sweep net (sprayed with a pyrethroid-based surface spray; Mortein Outdoor Barrier Surface Spray; Imiprothrin 0.3 g/Kg, 0.6 g/Kg Deltamethrin) collections performed daily by a single individual between 16:00–17:00 for a period of 2 d starting 24 h after the release of males. Sweep net collections were supplemented by Biogents Sentinel (operated continuously) [35] and Gravid *Aedes* Trap (GAT) [36] collections, with both traps being placed on the back patio. Captured mosquitoes were stored at -20 C until processed to determine the presence of rhodamine B as described above. Each subsequent release occurred 1 week after the prior release. During releases, temperatures ranged from 21.7–28.7 C and relative humidity ranged from 45–85% with no significant rain events. The owners of the residence gave permission for the study to be conducted on their property.

### Statistical analysis

Differences in male body staining, both visually and under fluorescence, seminal fluid marking, and persistence of male seminal fluid and body marking were analysed by two-way ANOVA with Tukey HSD post-hoc analysis. The data, represented as percentages, were arcsine transformed prior to analysis [37]. Differences in daily survivorship, determined by Kaplan-Meier survivorship curves, were analysed by the log-rank test [38]. Differences in mating competitiveness (i.e. proportion of females mated) was analysed using chi-square tests with the null hypothesis of 1:1 mating ratio. Mean comparisons of the proportions of males stained (arcsin transformed) between replicates exposed to and not exposed to additional sugar-feeding occurred using a t-test. Differences in male body staining (i.e. proportion of males stained) and seminal fluid marking (i.e. proportion of marked females that had mated with stained males) for the two treatment groups was analysed by the Fisher’s Exact Test with null hypotheses of 1:1 marking ratios. All statistical analyses were performed using the R (http://www.r-project.org/) and Prism (ver. 7.03; GraphPad) statistical software.

## Results

### Experiment 1: Effect of concentration and feeding time on male body and seminal fluid marking

**Fig 3:**
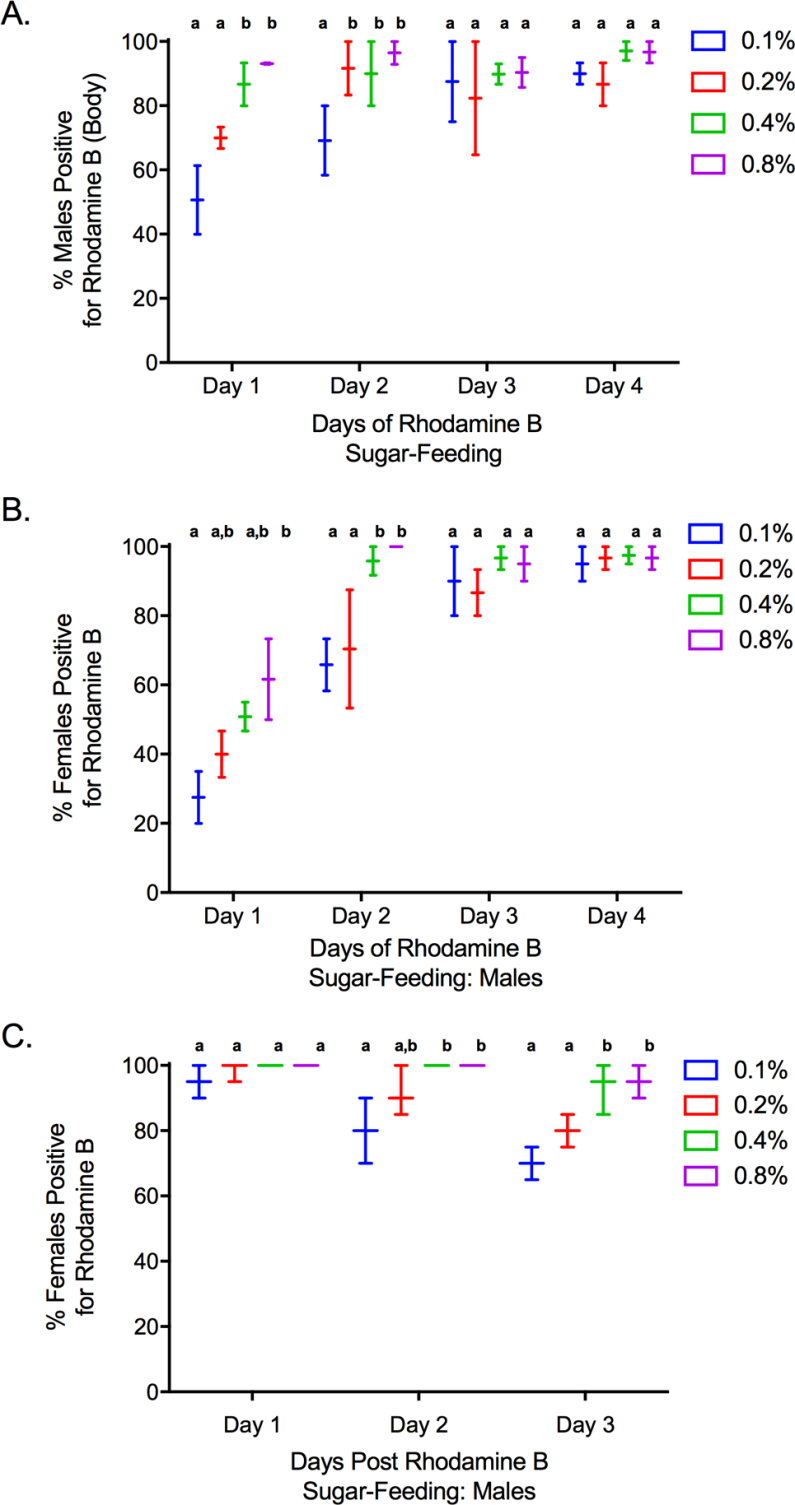
Effect of concentration and feeding time on male body and seminal fluid marking, transference to females after mating, and persistence of seminal fluid-marking after sugar-feeding. (**A**) Percent (mean±SE) of *Ae*. *aegypti* males with visual body marking after rhodamine B feeding at each concentration (w/v in 50% honey solution) when sugar-fed for 1–4 d. (**B**) Percent (mean±SE) of females positive for rhodamine B in the spermathecae, bursa or both after mating (24 h exposures) with males fed at each concentration (w/v in 50% honey solution) for 1–4 d. (**C**) Percent (mean±SE) of females positive for rhodamine B in the spermathecae, bursa or both after mating (24 h exposures) with males marked by rhodamine feeding (0.1–0.8% w/v for 4 d) and withheld from sugar-feeding for a period of 1–3 days.

There were significant effects of length of feeding (*F*_3,20_ = 3.34, *P =* 0.045) and concentration (*F*_3,20_ = 4.73, *P =* 0.02) on body and seminal fluid marking (Fig 3A; Table 1), with increases in both yielding the best marking results. Overall, 89.9±1.6% of males fed on 0.4% and 0.8% solutions were visually marked after 1 d of feeding, and 96.7±0.9% were marked after 4 d of feeding. When fed on 0.1% and 0.2% solutions, 60.3±3.6% were marked after 1 d of feeding and 88.3±1.6% were marked after 4 d of feeding. Similar effects of time (*F*_3,20_ = 43.9, *P*<0.001) and concentration (*F*_3,20_ = 5.95, *P =* 0.01) were observed for successful seminal fluid marking and transfer to females (Fig 3B; Table 1). Again, males fed on 0.4% and 0.8% produced the greatest seminal fluid marking, with 56.3±3.0% of females being positive for rhodamine in the bursa or spermathecae after exposure to males when those males were fed for 2 d, compared to 33.8±2.7% at 0.1% and 0.2%, respectively. After 4 d of male rhodamine feeding, there was no significant difference in seminal fluid marking across all treatments with 96.5±2.7% of females being positive for rhodamine B following male exposure.

**Table 1:** Efficacy of male body and seminal fluid staining as a function of concentration and length of feeding.

Percent of Males (mean ± SE)* with Visual Rhodamine B Staining (visible body staining)					
	Length of feeding	Day 1	Day 2	Day 3	Day 4
Rhodamine B Concentration	0.1%	50.7 (10.6)^a^	69.1 (10.8)^a^	87.5 (12.5)^a^	90.0 (3.3)^a^
	0.2%	70.0 (3.3)^a^	91.7 (8.3)^b^	82.4 (17.7)^a^	86.7 (6.7)^a^
	0.4%	86.7 (6.7)^b^	90.0 (10.0)^b^	89.8 (3.1)^a^	97.1 (2.9)^a^
	0.8%	93.1 (2.3)^b^	96.4 (3.6)^b^	90.4 (4.6)^a^	96.7 (3.3)^a^
Percent of Females (mean ± SE)* Positive for Rhodamine B after Mating (24 h exposure)					
	Length of feeding	Day 1	Day 2	Day 3	Day 4
Rhodamine B Concentration	0.1%	27.5 (7.5)^a^	65.8 (7.5)^a^	90.0 (10.0)^a^	95.0 (5.0)^a^
	0.2%	40.0 (6.7)^a,b^	70.4 (17.1)^a^	86.7 (6.7)^a^	96.7 (3.3)^a^
	0.4%	50.8 (4.2)^a,b^	95.8 (4.2)^b^	96.7 (3.3)^a^	97.5 (2.5)^a^
	0.8%	61.7 (11.7)^b^	100 (0)^b^	95.0 (5.0)^a^	96.7 (3.3)^a^

Summary of rhodamine B labelling of male *Ae*. *aegypti* body tissue and seminal fluid as a factor of length of sugar-feeding (1–4 d). Efficacy of male body labelling is represented as the percent (mean±SE) of males with visual body and efficacy of seminal fluid labelling is represented as the percent (mean±SE) of females positive for rhodamine B in the spermathecae and/or bursa after 24 h exposure to males at each time point.

* Different letters indicate statistically significant differences (p<0.05) among the rhodamine concentrations within each time treatment (day; going down columns).

### Experiment 2: Effect of rhodamine feeding on male survival

**Fig 4:**
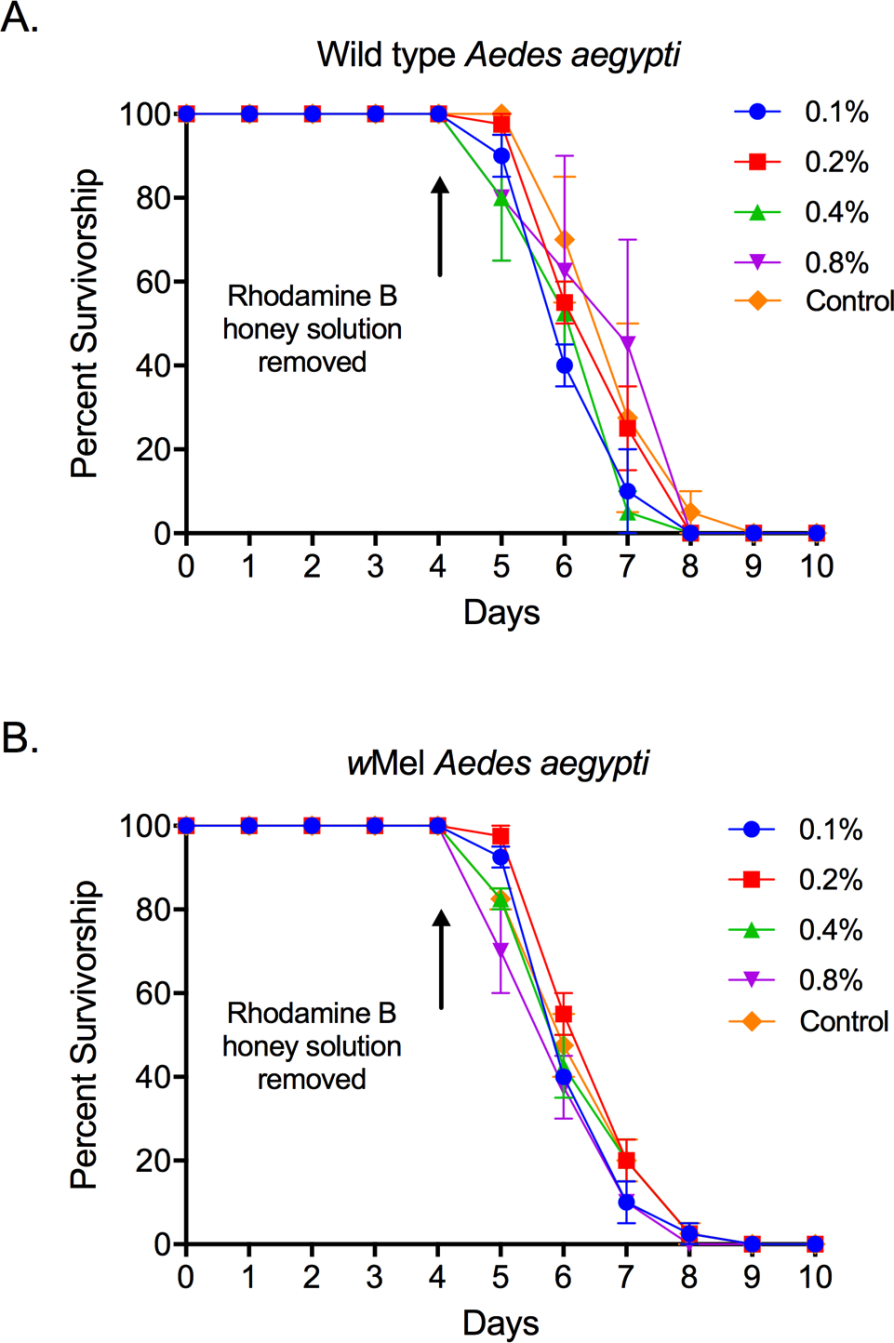
Male survivorship during and after rhodamine B feeding. Percent survivorship (mean±SE) of (**A**) wild type and (**B**) *w*Mel *Ae*. *aegypti* males during and after 4 d of feeding on 0.1, 0.2, 0.4, and 0.8% (w/v in 50% honey solution) rhodamine B solutions relative to unmarked controls (fed 50% honey).

No significant effect of rhodamine B feeding on survival was observed for either wild type (χ_1_ = 0.27, *P =* 0.60) or *w*Mel (χ_1_ = 0.28, *P =* 0.61) *Ae*. *aegypti* relative to honey-fed controls across all concentrations (Fig 4). No mortality was observed during honey-feeding and survival at the cessation of honey feeding among all concentrations and between the two strains was similar. Across all concentrations, the percent of wild type males surviving 1, 2, 3, and 4 d after cessation of honey-feeding as 86.9±5.0, 52.5±6.3, 21.3±8.1, and 1.8±0.9%. For *w*Mel *Ae*. *aegypti* males, survivorship 1, 2, 3, and 4 d after cessation of honey-feeding was 85.6±4.5, 43.7±3.4, 15.0±2.7, and 0%.

### Experiment 3: Persistence of rhodamine labelling of male body tissue and seminal fluid

There was a significant interaction between time since rhodamine feeding and concentration on the persistence of male body staining when scored visually (conspicuous body marking) (*F*_6,24_ = 17.13, *P*<0.001) and with fluorescence (*F*_6,24_ = 3.2, *P* = 0.02; Table 2). Specifically, increases in time since rhodamine feeding significantly decreased the percentage of males positive for the presence of rhodamine B across all concentrations, with the loss of the mark over time being greatest when males were fed on either a 0.1% or 0.2% solution. The percentage of males visibly positive for rhodamine in the 0.1% and 0.2% treatments dropped 65.8±3.0% percent (99.2±0.8 to 33.3±2.5%) between the 24 and 72 h treatments. In contrast, the percentage of males visibly positive for rhodamine in the 0.4% and 0.8% treatments dropped 22.5±2.1% (100±0 to 77.5±2.1%). Dissecting males and fluorescing the digestive tract greatly improved the percentage of males positive for rhodamine at 72 h post feeding. The percentage of males positive for rhodamine 72 h post sugar-feeding increased to 69.2±3.0% in the two lower treatments and to 84.2±1.5% in the higher treatments.

**Table 2:** Persistence of rhodamine B labelling of male seminal fluid and body tissue.

Percent of Females (mean ± SE)* Positive for Rhodamine B after 24 h Exposure to Males Withheld from Sugar-feeding for 24–72 h				
	Time Post Feeding	24 h	48 h	72 h
Rhodamine B Concentration	0.1%	95.0 (2.9)^a^	80.0 (5.8)^a^	70.0 (3.0)^a^
	0.2%	98.3 (1.7)^a^	91.7 (4.4)^a,b^	80.0 (2.9)^a^
	0.4%	100 (0)^a^	100 (0)^b^	93.3 (4.4)^b^
	0.8%	100 (0)^a^	100 (0)^b^	95.0 (2.9)^b^
Percent of Males (mean ± SE)* Positive for Rhodamine B: with Fluorescence				
	Time Post Feeding	24 h	48 h	72 h
Rhodamine B Concentration	0.1%	98.3 (1.7)^a^	81.7 (4.4)^a^	65.0 (2.9)^a^
	0.2%	100 (0)^a^	90.0 (2.9)^a,b^	73.3 (4.4)^a^
	0.4%	100 (0)^a^	96.7 (1.7)^b^	85.0 (2.9)^b^
	0.8%	100 (0)^a^	96.7 (1.7)^b^	83.3 (1.7)^b^
Percent of Males (mean ± SE)* Positive for Rhodamine B: Visible Body Staining				
	Time Post Feeding	24 h	48 h	72 h
Rhodamine B Concentration	0.1%	98.3 (1.7)^a^	70.0 (5.8)^a^	31.7 (4.4)^a^
	0.2%	100 (0)^a^	75.0 (2.9)^a^	35.0 (2.9)^a^
	0.4%	100 (0)^a^	93.3 (1.7)^b^	75.0 (2.9)^b^
	0.8%	100 (0)^a^	93.3 (4.4)^b^	80.0 (2.9)^b^

Summary of the persistence of rhodamine B staining of male *Ae*. *aegypti* seminal fluid and body tissue as a factor of time since feeding and concentration of rhodamine B. All males were fed on each concentration for a period of 4 d prior to the start of each trial.

* Different superscript letters indicate statistically significant differences (p<0.05) among the rhodamine concentrations within each time treatment (hour; down columns) treatment.

Similar to the persistence of male body staining, there was a significant interaction between time since rhodamine feeding and concentration (*F*_6,24_ = 2.80, *P =* 0.03) on the persistence of seminal fluid staining, scored as the presence/absence of rhodamine transferred to females during mating (Fig 3C; Table 2). Specifically, increases in time since rhodamine feeding significantly increased the percentage of mated females (i.e. sperm present) with no evidence of rhodamine B in the bursa and spermathecae when mated with males fed on either 0.1% or 0.2% rhodamine solutions. However, unlike male body staining, no significant effect of time since feeding was observed for seminal fluid labelling of males fed on 0.4% and 0.8% solutions. The percentage of females positive for rhodamine in the 0.1% and 0.2% treatments dropped 21.7±2.9% percent (96.7±1.6 to 75.0±2.9%) between the 24 and 72 h treatments. In contrast, the percentage of females positive for rhodamine in the 0.4 and 0.8% treatments dropped only 5.8±3.4% (100 to 94.2±2.4%).

### Experiment 4: Effect of additional sugar-feeding on male body and seminal fluid marking

**Fig 5:**
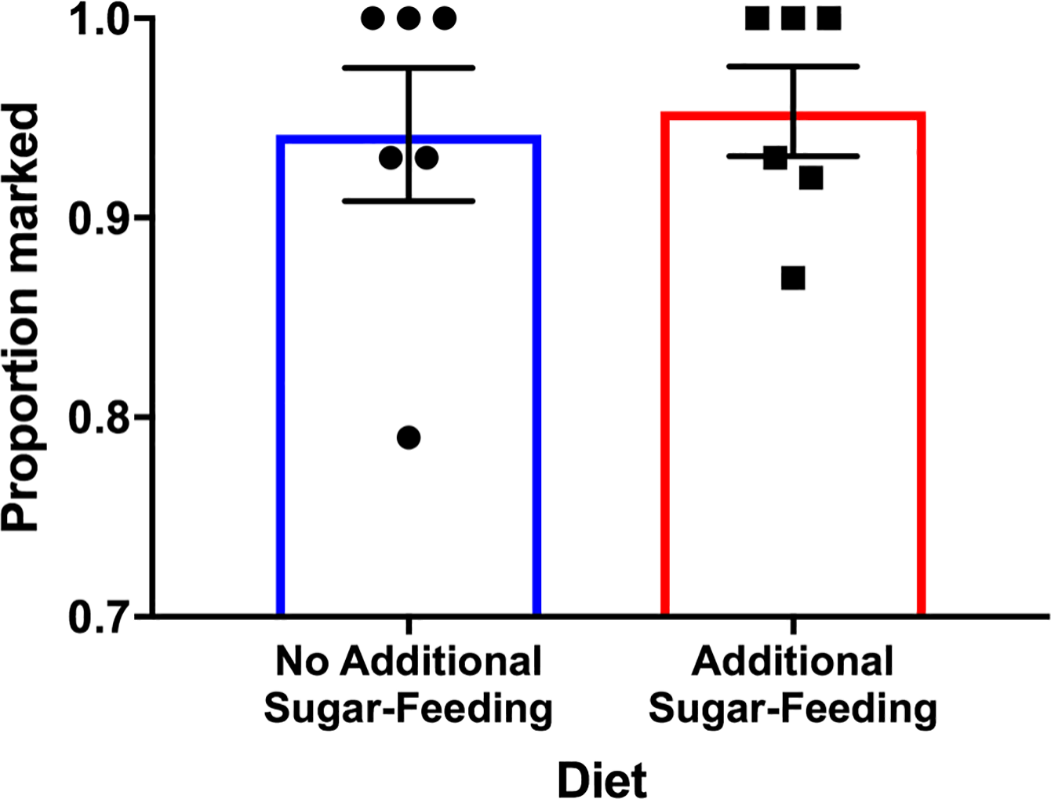
Effect of additional fluids on male body marking by rhodamine B. The proportion (mean ± SE) of male *Ae*. *aegypti* with visual body marking before (no additional sugar-feeding) and after the ingestion of an additional sugar meal (50% honey, no rhodamine B) 48 h after cessation of initial rhodamine feeding.

Trial one indicated that an additional feeding had no significant impact on male body marking (*t*_*10*_ = 0.16, *P =* 0.88; Fig 5). Overall, 94.2% (±3.3) of males were marked after 4 d of feeding on only the rhodamine (0.4%) solution and 95.3% (±2.2) of males were marked after 4 d of feeding on rhodamine (0.4%) then additional sugar-feeding (50% honey solution) 48 h later. Consistent with trial one, the results of trial 2 also suggested that there were no significant effects of additional sugar-feeding on body marking (Fisher’s Exact Test, *P =* 1) or seminal fluid marking (Fisher’s Exact Test, *P =* 0.39; Table 3). The amount of marking after 4 d of feeding was consistent with Experiment 3 with 98.3% of males exposed to 0.4% rhodamine solution marked 96 hours later and 96.8% of males exposed to 0.4% rhodamine as well as additional sugar-feeding (50% honey solution) marked 96 hours later. All males that had an additional honey feeding were noted to be engorged when knocked down. Of the females that had mated with stained males not exposed to additional sugar-feeding 83% displayed seminal fluid marking while 90% of females that mated with stained males exposed to additional sugar-feeding displayed seminal fluid marking under fluorescence (Table 3).

**Table 3:** Effect of secondary sugar-feeding on male body and seminal fluid marking.

Diet	Male Body Marked	Male Body Not Marked	Seminal Fluid Marked	Seminal Fluid Not Marked
No additional sugar-feeding	60	1	44	9
Additional sugar-feeding	61	2	47	5

Number of males *Ae*. *aegypti* with detectable body and seminal marking 96 h after feeding on rhodamine B (0.4%) for 4 days, with or without additional fluid intake during secondary sugar-feeding (50% honey, no rhodamine B). Assessment of seminal fluid marking was determined by the presence or absence of rhodamine B in the bursa and/or spermathecae of females allowed to mate with an individual male.

### Experiment 5: Mating competitiveness of rhodamine B marked males under semi-field conditions

**Fig 6:**
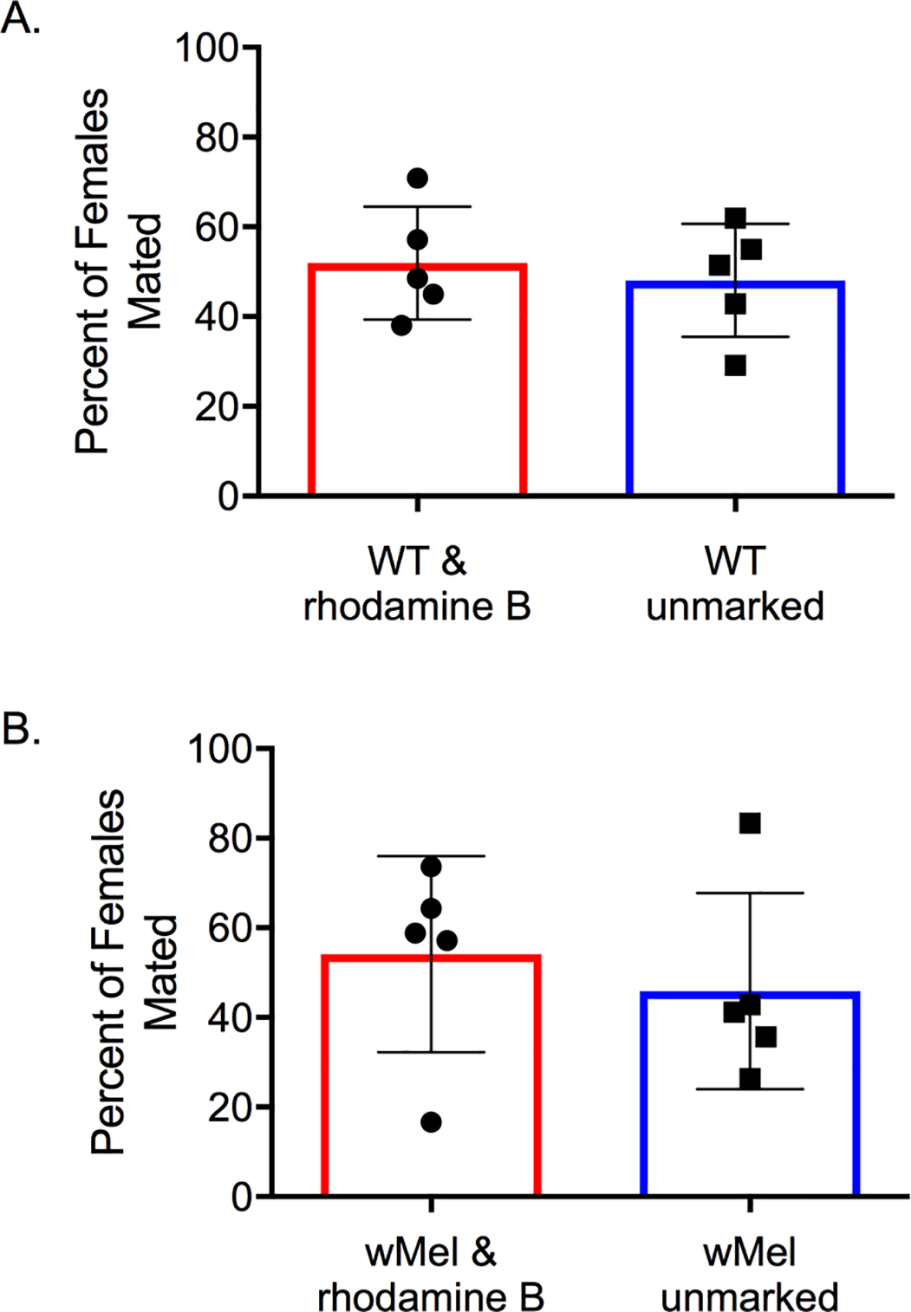
Mating competitiveness of rhodamine B marked males. Results of mating competitiveness assays represented as the percent (mean±SE) of females mated by (**A**) wild type and (**B**) *w*Mel marked *Ae*. *aegypti* males vs. unmarked controls. Columns represent treatment means and points represent observations from individual replicates during which 80 virgin females (3–5 d old) were introduced to and kept in the presence of 40 virgin marked and 40 unmarked males (4–5 d old) in a semi-field cage for 72 h.

No significant effect of rhodamine B feeding on male competitiveness was observed for either wild type (χ_1_ = 2.56, *P =* 0.11) or *w*Mel (χ_1_ = 0.64, *P =* 0.42) *Ae*. *aegypti* under semi-field conditions (Fig 6). The mean (±SE) percent of females mated by rhodamine marked wild type and *w*Mel *Ae*. *aegypti* males was 51.9±12.6% and 54.1±9.7%, respectively, when competing against unmarked controls.

### Experiment 6: Rhodamine B transfer to wild females during a field release

Over the three field releases, 31.7±12.8% of collected males (total collected = 53) and 27.7±12.5% of captured females (total collected = 32) were rhodamine B positive (Table 4), demonstrating successful transfer of rhodamine B from marked males under normal field conditions and successful recapture of marked males. 38.0±13.6% of captured females were mated by wild males (sperm, no rhodamine) and 34.3±1.7% were unmated.

**Table 4:** Results of field releases of rhodamine B marked males.

	Males: Rhodamine Pos.	Males: Unmarked	Females: Rhodamine Pos.	Females: Wild mated (sperm, no rhodamine)	Females: Virgin (no sperm or rhodamine)
Release 1	10 (34.5%)	19 (65.5%)	4 (36.4%)	3 (27.3%)	4 (36.4%)
Release 2	3 (17.6%)	14 (82.4%)	2 (13.3%)	8 (53.3%)	5 (33.3%)
Release 3	3 (42.9%)	4 (57.1%)	2 (33.3%)	2 (33.3%)	2 (33.3%)
Mean (SD)	5.3±4.0 (31.7±12.8%)	12.3±7.6 (68.3±12.8%)	2.7±1.2 (27.7±12.5%)	4.3±3.2 (38.0±13.6%)	3.7±1.5 (34.3±1.7%)

Number (% of total) of rhodamine B marked and unmarked males and females collected during each 72 hr MRR experiment. Rhodamine B present in females was transferred during mating with released rhodamine marked males. Released males were marked by feeding on a 0.4% (w/v in 50% honey) rhodamine B solution for a period of 4 d beginning 12–24 hr after eclosion from the pupal case.

## Discussion

Mark-release-recapture experiments are vital to our understanding of mosquito vector populations, disease transmission dynamics, and efficacy of control interventions [7]. Recent interest in SIT/IIT-based control initiatives to combat dengue, Zika and other mosquito-borne diseases [39, 40] has highlighted the need for male-focused MRR experiments, experiments that would be enhanced by the development of novel marking techniques that enable operators to assess the efficacy of intervention strategies quickly and easily [41]. Our results support the use of rhodamine B, a fluorescent powder administered through sugar-feeding, as one such marking technique. Rhodamine B sugar-feeding successfully marked the body and seminal fluid of wild-type and *w*Mel *Ae*. *aegypti*, both lasting over the reported life expectancy of released males [42–44] post sugar-feeding and without significantly impacting survival. Overall, transfer of marked seminal fluid occurred at rates >95% post sugar-feeding with no impact on male competitiveness or survivorship relative to unmarked individuals. Importantly, rhodamine B transfer to wild females was observed during multiple field releases as well as the recapture of marked males. These results reveal rhodamine B marking to be a potentially useful tool to for SIT/IIT programs to measure male mating efficacy, as well as a viable body marking technique for male-based MRR experiments, and, to the best of our knowledge, is the only technique that allows both to be accomplished using the same marking process. As a standalone method for use in mating competitiveness assays, rhodamine B marking is advantageous to costly PCR and stable isotope marking methods [16, 45, 46], as well as time consuming female fertility assays used to assess competitiveness of sterilised males [47–49].

A potential limitation of rhodamine B marking via sugar-feeding is that longevity is presumably contingent upon males not sugar-feeding soon after release as ingestion of additional fluids will likely dilute the mark. However, as we have demonstrated, the ingestion of additional fluids during sugar-feeding after initial marking did not have a negative effect on male body or seminal fluid marking, at least after a single sugar-feeding. Additionally, in the case of *Ae*. *aegypti*, sugar-feeding does not appear to be a priority for females [50, 51] and, although there is little data available for males, these observations are supported by a recent report by Fikrig et al. [52] highlighting poor male sugar-feeding under free-flight conditions. These observations, combined with the knowledge that the mark persists through at least one additional sugar-feeding, indicate that the efficacy of this method will remain high during the first 1–3 days of release. After this time, recently derived male daily survival probabilities (0.48–0.82 [42–44, 53]) suggest the number of released males able to mate with wild females will be greatly reduced. Nonetheless, knowledge of male sugar-feeding in the wild is still poorly understood and further investigations into the effect of multiple sugar-feedings on male marking and survival are warranted. Thus, the best application of this marking technique as assessed is to track the mating success of released males relative to wild males during operational release timelines in a defined release site.

Cost and ease of use are important attributes for a marker [6], particularly when considering the cost and logistics of treating 1000s of individuals. The cost of producing a litre of rhodamine B-treated honey was $1.40 (0.4% w/v), which is sufficient to treat ≥2,500 males. Additionally, as others have noted [54], this method is easy to implement for feeding hundreds of insects at once as it requires no special skills or equipment to administer. Although treatment costs are minimal, this method requires fluorescent microscopy equipment (e.g. microscope, illuminator, filters), which in this study cost ca. $11,000 USD (Scope Scientific Pty Ltd, Queensland, Australia). Despite these significant upfront costs, per sample analysis and treatment costs are marginal relative to alternative methods allowing researchers to recoup upfront costs. For example, Hamer et al. [15] detail treatment costs of $1.50 and $3.69 per 100 larvae for ^15^N and ^13^C enrichment, respectively, and $7 per sample (1–5 individuals) analysis costs (dual^15^N and ^13^C mass spectrometry analysis). Further, the time required for determining the presence/absence of rhodamine B is <30 min once staff have acquired the appropriate dissection and microscopy skills, whereas stable isotope analysis may range anywhere from 1 to 20 weeks depending on the facility and expense of rapid analysis. Of note, it is important to acknowledge that rhodamine B is a notifiable chemical with limited evidence of carcinogenicity in animals and no adequate data for humans [55]. It is equally important to acknowledge that it has long been used as a biomarker in wildlife studies [56–58] and lethal dose calculations for a variety of invertebrates and vertebrates are relatively high [57], well above what an individual mosquito could ingest (<2 mg) during a single sugar meal (max volume 0.4 μl [59]). At these amounts rhodamine B poses little health risk to humans or species that may consume released mosquitoes; however, best practice dictates that a full assessment of the risk of rhodamine B in this manner be performed by appropriate institutional research review boards and ethics committees prior to initiation of field and laboratory studies.

In summary, this study reveals rhodamine B labelling to be an economical and rapid evaluation method for male-based control strategies as well as a potential body marking technique for male focused MRR experiments. The use of this method has the potential to enable operators to determine the efficacy of their control method quickly and easily, allowing them to optimise releases to achieve optimal population suppression. These observations are bound by the limitations in extending laboratory observations as a prediction of what to expect in the field and we strongly recommend that small-scale field MRR experiments be performed to obtain more accurate estimates of male survival and mark persistence prior to adoption for operational assessments. These studies would benefit from comparisons to classical marking techniques such as fluorescent powders to better understand the utility of rhodamine B body marking in male-focused survival and dispersal studies.
